# Transanal repair of anastomotic leakage after oncologic low anterior resection: a prospective cohort

**DOI:** 10.1007/s10151-024-03103-1

**Published:** 2025-02-14

**Authors:** W. Lossius, T. Stornes, T. E. Bernstein, A. Wibe

**Affiliations:** 1 Department of Surgery, St. Olav’s University Hospital, Trondheim University Hospital, Postboks 3250 Torgarden, 7006 Trondheim, Norway; 2 Norwegian Research Center for Minimally Invasive and Image-guided Diagnostics and Therapy, St. Olav’s Hospital, Trondheim University Hospital, Trondheim, Norway; 3 Institute of Clinical and Molecular Medicine, Norwegian University of Science and Technology, Trondheim, Norway

**Keywords:** Transanal repair, Anastomotic leak*, Rectal cancer, Low anterior resection

## Abstract

**Background:**

Anastomotic leakage is a common complication after low anterior resection for rectal cancer, often resulting in a permanent stoma. This study aimed to evaluate the effectiveness of early detection, sepsis control, and transanal repair in managing anastomotic leakage.

**Methods:**

In this prospective cohort study conducted from January 2018 to June 2022 at a Norwegian university hospital, patients undergoing resectional surgery for rectal cancer were assessed for anastomotic leaks. Early detection involved CT with rectal contrast and flexible endoscopy. Repair eligibility required involvement of less than half the anastomotic circumference and no ischemia or retraction of the colon. The cavity outside the anastomotic defect was cleaned using a catheter for intermittent irrigation or endoluminal vacuum therapy. A diverting stoma was created, and a transabdominal pelvic drain was inserted if not already present. Once sepsis was controlled and the cavity was clean, the defect was sutured using a transanal minimally invasive surgery access platform or an open transanal technique, based on anastomosis level. Healing was confirmed via computed tomography (CT) with rectal contrast and rigid proctoscopy before reversing diverting stomas, and again at 12 months. A supplementary video demonstrates the technique.

**Results:**

Of 22 identified anastomotic leaks, 11 underwent transanal repair, resulting in healed anastomosis for nine patients and restored bowel continuity for eight. Among these, five reported major low anterior resection syndrome. Median hospital stay was 20 days, with no 90-day mortality.

**Conclusions:**

This anastomosis-preserving approach for treating anastomotic leakage shows promise, potentially preserving bowel function and reducing permanent stoma rates.

**Supplementary Information:**

The online version contains supplementary material available at 10.1007/s10151-024-03103-1.

## Introduction

### Background and rationale

Anastomotic leakage (AL) is a feared complication of low anterior resection for rectal cancer. While reported incidence varies from 2% to 28% or more, larger studies and meta-analysis estimate that AL occurs in about 10% of procedures [1–3] and with 30-day mortality of 2.6% [3]. Handling this complication aims to treat sepsis, minimize the rate of permanent stoma for the affected patients, and preserve adequate rectal function and quality of life. Transanal suture is an established option for anastomotic leakage of ileoanal pouch reservoirs at our center. However, while some results have been promising, its use for leakage of colorectal anastomoses is not widely established [4]. This may be due to technical difficulties in gaining adequate exposure to the anastomosis, as stapled colorectal anastomoses are likely to be higher and thus more difficult to reach. Other complicating factors may include differences in the healing of the small bowel versus the large bowel and possible exposure to neoadjuvant radiation. Experience from transanal minimally invasive surgery (TAMIS) for resection of advanced rectal adenomas and early rectal cancers, while alleviating exposure challenges, allows for a systematic approach to the transanal repair of anastomotic leakage [5].

### Objectives

This single-center study presents the surgical method and results of an anastomosis-preserving approach to handling anastomotic leakage after low anterior resection. It applied a protocol of early detection, irrigation, and drainage, and a temporary diverting stoma, followed by secondary transanal suture using either a transanal minimally invasive surgery access platform or conventional retractors, as appropriate. Primary endpoints were fully healed anastomosis and restoration of bowel continuity, and secondary endpoints were length of stay, 90-day mortality, and functional outcomes measured by the low anterior resection syndrome (LARS) score [6].

## Patients and methods

### Study design

Cohort observational study with supplementary video.

### Setting

This clinical study took place at St. Olav’s University Hospital in Trondheim, Norway.

### Participants

All patients treated by low anterior resection for rectal cancer between January 2018 to June 2022 were included. Patients suffering an anastomotic leakage who were treated by secondary transanal suture were asked to provide informed consent and complete LARS surveys [6] with the assistance of a study nurse. Passive opt-out consent was obtained for the remaining patients. The study was approved by the Regional Committee for Medical and Health Research Ethics Central Norway.

### The procedure

**Fig. 1:**
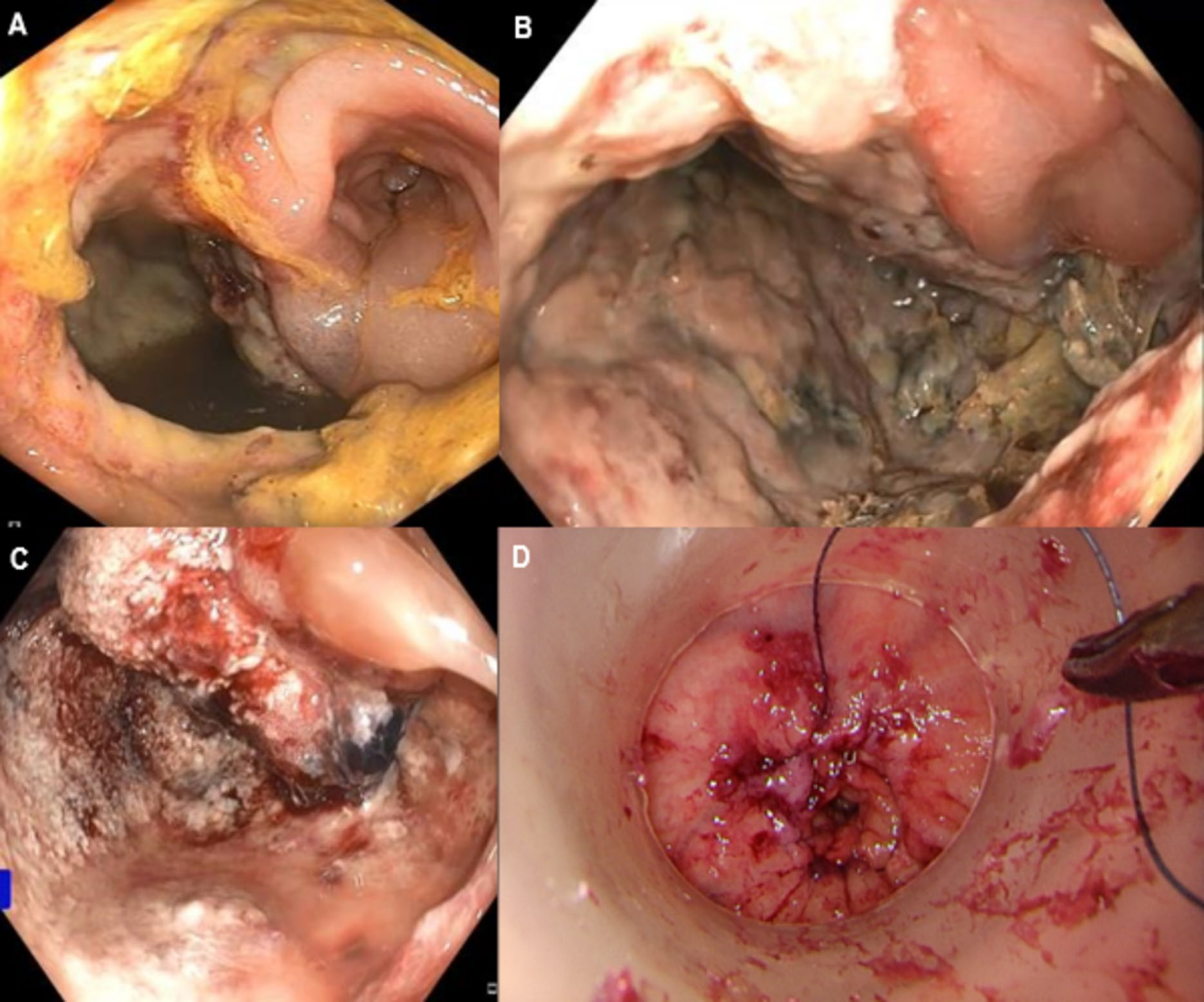
Transanal repair. **A** Anastomotic leak upon first examination, dehiscent from 6 to 10 o’clock (POD 12). **B** After first EVT (POD 15). **C** After second EVT (POD 20). **D** Transanal repair by TAMIS (POD 23). *POD* postoperative day, *EVT* endoluminal vacuum therapy

The treatment leading up to and including the primary surgical procedure was in accordance with national guidelines [7]. Postoperative day 2–3 body temperature ≥ 38.0 °C or highly elevated CRP (above 100 mg/L for laparoscopic procedure and 150 mg/L for an open procedure), in presence of clinical suspicion of anastomotic leakage (e.g., more pain than expected) were followed by a CT scan with rectal contrast and by flexible endoscopy. Elevated CRP levels without clinical signs implied repeated measurements the next day and examination of the anastomosis if there was a lack of improvement. The anastomosis was likewise examined in the presence of a clinical suspicion, without abnormal CRP elevation. Anastomotic leakage was deemed eligible for repair if the defect involved less than half of the circumference and there was no evidence of ischemia or retraction of the colon. The cavity behind the anastomotic defect was then irrigated, and an endoluminal device (Endo-SPONGE^®^, Braun Surgical S.A., Barcelona, Spain) for continuous vacuum therapy (if relatively large defect) or a 12Fr silicone catheter was placed through the defect for intermittent irrigation (20 mL × 3 per day). If a transabdominal drain or diverting stoma was not present from the primary surgery, a drain was placed and a diverting stoma constructed during this procedure. If the pelvis was not accessible transabdominally, a perianastomotic drain was placed upon suturing. Secondary diversion was preferentially performed on the left colon, given adequate mobility, to facilitate easy irrigation. Patients were treated by intravenous tazobactam + piperacillin at a dose of 4 g , administered three times (iv). The anastomosis was examined again every 3–4 days, and when the cavity was clean and the sepsis was under control, the defect was sutured. Depending on the level of anastomosis, an open transanal approach or a transanal minimally invasive surgery access platform (The GelPOINT Path transanal access platform^®^, Applied Medical, Rancho Santa Margarita, CA, USA) was used, applying either interrupted vicryl 3–0 sutures or a continous v-loc 90 3–0 suture (Fig. 1 and Video 1). Intravenous antibiotics were continued for 5–14 days postoperatively, until CRP was normalized. A CT scan with rectal contrast and rigid proctoscopy was performed after 3 months, and again at 6 months, if necessary, to ensure successful healing before restoring continuity. A repeated CT scan was done at 12 months.

### Statistical methods

Categorical variables are described as frequencies and percentages; parametrically distributed data are presented as means with standard deviations, and non-parametrically distributed data as medians with percentile quartiles. ANOVA, independent-sample *t*-test, chi-squared, Mann–Whitney, or Fisher’s exact were used for comparison between groups, as appropriate. Statistical analyses were performed with IBM SPSS Statistics for Windows, version 29.0.1.0 (Armonk, NY; IBM Corp).

## Results

**Fig. 2:**
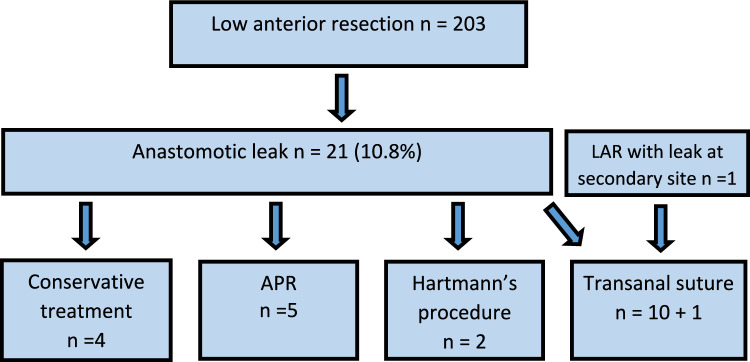
Treatment of 22 anastomotic leaks following low anterior resection for rectal cancer. *LAR* low anterior resection, *APR* abdominoperineal resection

We recognized 21 anastomotic leaks at the primary center, and 10 of these were treated with transanal suture. In addition, one patient with an anastomotic leak from a secondary center was transferred and treated with the same protocol, for a total of 11 patients receiving transanal suture. Two patients opted out of consent. The flowchart (Fig. 2) shows the total number of participating patients.

Baseline characteristics of all low anterior resections at the study center are presented in Table 1, and the results of treatment of all anastomotic leaks (including the patient transferred from a secondary center) are given in Table 2. There was a 3.4% 90-day mortality for all low anterior rectal resections, but no 90-day mortality in the group suffering anastomotic leakage. One patient with transanal repair healed with stenosis and required endoscopical dilatation before successful restoration of bowel continuity.

**Table 1:** Baseline characteristics of low anterior resections at primary center

	All LAR	No leakage	Leakage	*P*
	203	182 (89.7%)	21 (10.3%)	
Sex				
Male	122 (60.1%)	106 (58.2%)	16 (76.2%)	0.16
Female	81 (39.9%)	76 (41.8%)	5 (23.8%)	
Age, years (mean, SD)	65.5 (10.8)	66.6 (10.7)	65.5 (12.6)	0.67
ASA				
1–2	121 (56.9%)	110 (60.4%)	11 (52.4%)	0.63
3	75 (36.9%)	66 (36.3%)	9 (42.9%)	
4	7 (3.4%)	6 (3.3%)	1 (4.8%)	
BMI (median, IQR)	25.2 (23.1–28.0)	25.2 (23.1–27.8)	25.1 (22.9–28.4)	0.91
Tumor level				
≤ 7 cm	19 (9.4%)	18 (9.9%)	1 (4.8%)	0.70
> 7 cm	183 (90.6%	164 (90.1%)	20 (95.2%)	
cT-status				
1	16 (7.9%)	14 (7.7%)	2 (9.5%)	0.60
2	55 (27.1%)	51 (28.0%)	4 (19.0%)	
3	94 (46.3%)	85 (46.7%)	9 (42.9%)	
4a	31 (15.3%)	26 (14.3%)	5 (23.8%)	
4b	7 (3.4%)	6 (3.3%)	1 (4.8%)	
cN-status				
0	113 (55.7%)	99 (54.4%)	14 (66.7%)	0.16
1	49 (24.1%)	43 (23.6%)	6 (28.6%)	
2	41 (20.2%)	40 (22.0%)	1 (4.8%)	
cM-status				
0	173 (85.2%)	154 (84.6%)	19 (90.5%)	0.75
1	30 (14.8%)	28 (15.4%)	2 (9.5%)	
Neoadjuvant radiotherapy				
No	128 (63.1%)	113 (62.1%)	15 (74.4%)	0.48
Yes	75 (36.9%)	69 (37.9%)	6 (28.6%)	
Technique				
Open	44 (21.7%)	38 (20.9%)	6 (28.6%)	0.71
Lapsc	140 (69.0%)	126 (69.2%)	14 (66.7%)	
RAL	19 (9.4%)	18 (9.9%)	1 (4.8%)	
Diverting stoma				
No	60 (29.6%)	53 (29.1%)	7 (33.3%)	0.80
Yes	143 (70.4%)	129 (70.9%)	14 (66.7%)	

*LAR* low anterior resection, *RAL* robot-assisted laparoscopic, SD standard deviation, IQR interquartile range

**Table 2:** Treatment of all anastomotic leaks

	Transanal suture	Conservative, APR or HP
*n*	11	11
ASA 2	6 (54.5%)	7 (63.6%)
ASA 3	5 (45.5%)	4 (36.4%)
Age, years^a^	68 (50–72)	69 (57–72)
Tumor level^a^	11 (10–15)	12 (10–14)
Neoadjuvant radiotherapy	2 (18.2%)	4 (36.4%)
No neoadj. radiotherapy	9 (81.8%)	7 (63.6%)
Length of stay^a^	20 (17–32)	15 (7–17)
POD of first procedure^a^	4 (4–7)	
Number of procedures^a^	2 (2–4)	
Healed at 3 months	9 (81.8%)	
Healed at 6 months	10 (90.9%)	
Healed at 12 months	9 (81.8%)^b^	
Healed with bowel continuity	8 (72.7%)^c^	
Weeks with stoma^a^	32 (19–40)	
Stoma free at end of observation	8 (72.7%)	3 (27.3%)
LARS score^a^	32 (4–35)	
Months from stoma closure to LARS score^a^	40 (12–59)	

*HP* Hartmann’s procedure; *POD* postoperative day

^a^Median (interquartile range)

^b^One patient proved to have persistent AL after stoma closure

^c^In one patient with a healed anastomosis, the stoma was never closed owing to progressive metastatic disease

### The 11 anastomotic leaks in which transanal repair was not attempted

Among these, four patients were not initially recognized as having anastomotic leaks or examined according to protocol. One patient was treated with antibiotics for an unclear focus, as the CT scan was negative; therefore, the procedure for endoscopic inspection was not followed, but his condition was recognized on later controls. Another had negative CT and inspection (bleeding, but no recognized defect), and was treated by antibiotics; CT before stoma reversal demonstrated a leak. The third patient was recognized as likely having a minimal leak, but procedure was not adhered to, and the patient was treated with antibiotics without examination by inspection or CT. The leak was confirmed upon examination, before reversal of stoma. The last patient showed no signs of anastomotic leak, nor were any recognized before stoma reversal, but it was demonstrated upon the first control after reversal of stoma. All of these had a divertive loop ileostomy as part of the initial procedure and were assigned to conservative management.

Three patients had dehiscence of more than half of the circumference, two had severe sepsis with widespread abdominal contamination, one had ischemia of the afferent colon, and one developed severe colitis and was later diagnosed with ulcerative colitis. Endoluminal vacuum therapy (EVT) was initiated for this patient; however, it was discontinued due to the development of severe colitis. Another patient, who had undergone multivisceral surgery including cystoprostatectomy, also began EVT. However, the attempt was abandoned after a single session, as the defect was deemed too large. EVT was not initiated for any of the other leaks that were not treated with transanal repair.

Three out of eight patients (37.5%) were successfully repaired, and with reestablished bowel continuity, reported no LARS, but major LARS was reported by the remaining five patients (62.5%).

## Discussion

We found that half of our anastomotic leak patients were eligible for transanal repair. Healing of the anastomosis was obtained in 9 out of 11 (81.8%) patients, of whom bowel continuity was reestablished in 8 (72.7%). By comparison, the CLEAN study achieved respective numbers of 70% and 67% in a population with a higher percentage of neoadjuvant radiotherapy (73%, compared with 29% in the present study) [4]. While Table 2 is not meant to directly compare the different approaches to AL, it demonstrates that there were no major differences in the selection of approach (age, ASA, tumor level, and neoadjuvant therapy) apart from the criteria described in the methodology. All the patients with undetected early leaks had divertive stomas as part of the primary procedure. Strict adherence to protocol could potentially have identified and allowed transanal repair of two of these, possibly three if subjected to repeated inspection. The fourth was only recognized after reestablishment of bowel continuity, demonstrating that divertive stomas may mask anastomotic leaks.

While the present study is small, the prevalence of major LARS (62.3%) in the limited study group seems comparable to the prevalence seen in a European cross-sectional study, reporting major LARS in 61.8%, minor LARS in 25.8%, and no LARS in only 12.5% of patients after low anterior resection [8]. This suggests an acceptable functional outcome of performing transanal repair on eligible patients.

Managing anastomotic leaks prior to this study involved Hartmann’s procedure, abdominoperineal resections, percutaneous drainage, as well as isolated endoluminal vacuum therapy. Before starting this project, the prevailing opinion, at least at our center, was that, in case of an anastomotic leak, septic control and bad functional outcomes closed the door for a sphincter-preserving approach. However, clinical routines for early diagnosis of leaks, new approaches for drainage and irrigation of the abscess, as well as innovative surgical techniques for closing the defect imply that some patients with an anastomotic leak may avoid a permanent stoma and retain acceptable bowel function.

The moderate length of stay and the zero 90-day mortality might be a measure of the method’s ability to gain septic control. While anastomotic leakage did increase length of stay in the transanal suture approach compared with other approaches, an additional 5 days in the hospital may be acceptable when the alternative is a permanent stoma.

### Strengths and weaknesses

While the current study is of limited size, its prospective nature allowed a high degree of completeness of data, and apart from a few research centers, publications of this type of procedure are limited to case reports, suggesting limited use. The present data describe everyday practice at a modern surgical unit of comparable size to many centers. Both the detailed description of the procedure and the supplementary video should allow for further spread of the use of this approach. As an observational study, it is by nature susceptible to bias regarding the selection of patients and interpretation of the results. The use of objective methods, such as CT with rectal contrast to confirm healing of the anastomosis, and the involvement of an independent scientific nurse in obtaining the functional results, aimed to reduce bias.

## Supplementary Information

Below is the link to the electronic supplementary material.

Supplementary File 1 (MOV 104653 KB)

## Data Availability

No datasets were generated or analyzed during the current study.
